# Integrin-targeted quantitative optoacoustic imaging with MRI correlation for monitoring a BRAF/MEK inhibitor combination therapy in a murine model of human melanoma

**DOI:** 10.1371/journal.pone.0204930

**Published:** 2018-10-03

**Authors:** Philipp M. Kazmierczak, Neal C. Burton, Georg Keinrath, Heidrun Hirner-Eppeneder, Moritz J. Schneider, Ralf S. Eschbach, Maurice Heimer, Olga Solyanik, Andrei Todica, Maximilian F. Reiser, Jens Ricke, Clemens C. Cyran

**Affiliations:** 1 Department of Radiology, Laboratory for Experimental Radiology, University Hospital, LMU Munich, München, Germany; 2 iThera Medical GmbH, München, Germany; 3 Comprehensive Pneumology Center, German Center for Lung Research, Munich, Germany; 4 Department of Nuclear Medicine, University Hospital, LMU Munich, München, Germany; University of Queensland Diamantina Institute, AUSTRALIA

## Abstract

**Purpose:**

To investigate α_v_β_3_-integrin-targeted optoacoustic imaging and MRI for monitoring a BRAF/MEK inhibitor combination therapy in a murine model of human melanoma.

**Materials and methods:**

Human BRAF V600E-positive melanoma xenograft (A375)-bearing *Balb/c* nude mice (n = 10) were imaged before (day 0) and after (day 7) a BRAF/MEK inhibitor combination therapy (encorafenib, 1.3 mg/kg/d; binimetinib, 0.6 mg/kg/d, n = 5) or placebo (n = 5), respectively. Optoacoustic imaging was performed on a preclinical system unenhanced and 5 h after i. v. injection of an α_v_β_3_-integrin-targeted fluorescent probe. The α_v_β_3_-integrin-specific tumor signal was derived by spectral unmixing. For morphology-based tumor response assessments, T2w MRI data sets were acquired on a clinical 3 Tesla scanner. The imaging results were validated by multiparametric immunohistochemistry (ß3 –integrin expression, CD31 –microvascular density, Ki-67 –proliferation).

**Results:**

The α_v_β_3_-integrin-specific tumor signal was significantly reduced under therapy, showing a unidirectional decline in all animals (from 7.98±2.22 to 1.67±1.30; p = 0.043). No significant signal change was observed in the control group (from 6.60±6.51 to 3.67±1.93; p = 0.500). Immunohistochemistry revealed a significantly lower integrin expression (ß_3_: 0.20±0.02 vs. 0.39±0.05; p = 0.008) and microvascular density (CD31: 119±15 vs. 292±49; p = 0.008) in the therapy group. Tumor volumes increased with no significant intergroup difference (therapy: +107±42 mm^3^; control +112±44mm^3^, p = 0.841). In vivo blocking studies with α_v_β_3_-integrin antagonist cilengitide confirmed the target specificity of the fluorescent probe.

**Conclusions:**

α_v_β_3_-integrin-targeted optoacoustic imaging allowed for the early non-invasive monitoring of a BRAF/MEK inhibitor combination therapy in a murine model of human melanoma, adding molecular information on tumor receptor status to morphology-based tumor response criteria.

## Introduction

Overactivation of the mitogen-activated protein kinase (MAPK) signal pathway by b-rapidly accelerated fibrosarcoma (BRAF) gene mutations V600E/K leads to uncontrolled proliferation of human cells and is a central mechanism of oncogenesis in melanoma [1, 2]. Selective BRAF inhibitors (BRAFi) disrupt this oncogenic stimulus and demonstrate high initial tumor response rates in metastatic melanoma [3, 4]. However, intrinsic or acquired BRAFi resistance limits long-term tumor response to BRAFi monotherapies [5]. One major mechanism of acquired BRAFi resistance is MAPK pathway activation by the mitogen-activated extracellular signal-regulated kinase (MEK), which may be overcome by selective MEK inhibitors (MEKi) [6]. Dual targeting of the MAPK signal pathway by a BRAFi/MEKi combination therapy demonstrated significantly improved overall and progression-free survival in patients with advanced BRAF-mutant melanoma compared to BRAFi monotherapy [7]. BRAFi/MEKi combination therapy is a first-line option in patients with BRAF-mutant metastatic melanoma (National Comprehensive Cancer Network Guidelines Version 1.2017, www.nccn.org).

Targeted therapies yield only subtle effects on tumor size and therefore limit the applicability of morphology-based criteria of tumor response [8]. As functional and molecular imaging allow for the non-invasive characterization of the tumor microenvironment beyond morphology, they bear the potential to provide novel, complementary imaging biomarkers of tumor response [9]. Functional and molecular imaging biomarkers may be better suited than size-based response criteria in correlation with clinical endpoints such as early therapy response or progression-free survival [10, 11].

α_v_ß_3_-integrin is a transmembrane protein overexpressed on angiogenic endothelium and tumor cells [12]. Depending on the investigated tumor model, α_v_ß_3_-integrin is a target structure for the non-invasive in vivo investigation of tumor angiogenesis and tumor cell populations [13]. In melanoma, α_v_ß_3_-integrin plays an important role in neoangiogenesis and tumor progression from the non-invasive, radial to the invasive, vertical growth phase [14, 15]. Herzog et al. demonstrated the applicability of optoacoustic imaging with a targeted fluorescent probe for the characterization of α_v_ß_3_-integrin receptor status in human tumor xenografts in vivo [16].

The purpose of this experimental proof-of-principle study was to investigate α_v_ß_3_-integrin-targeted optoacoustic imaging and MRI for the non-invasive in vivo monitoring of a BRAFi/MEKi combination therapy in a murine xenograft model of human melanoma, validated by multiparametric ex vivo immunohistochemistry. We hypothesized that the α_v_ß_3_-integrin-specific optoacoustic signal would be significantly reduced under targeted therapy, delivering a surrogate of suppressed tumor α_v_ß_3_-integrin expression over the treatment course and adding quantitative, dual time point molecular information on the tumor microenvironment to morphology-based assessments of tumor response.

## Materials and methods

The study was approved by the Government of Upper Bavaria Committee of Animal Research (Gz. ROB-55.2-2532.Vet_02-15-204) and conducted in accordance with the National Institutes of Health Guide for the Care and Use of Laboratory Animals. All applicable institutional and/or national guidelines for the care and use of animals were followed. Every effort was taken to reduce animal suffering. We kept the mice in individually ventilated cages (n = 4 mice per cage, relative air humidity 65% at n = 18 room air changes/h, temperature 26°C, light-dark-cycle 12 h), nourished with water and small animal nutrition. Nest boxes and nestles ensured environmental enrichment. Daily animal monitoring including weighing and tumor growth measurement was conducted. Abnormal inactivity was considered an indicator of pain and was treated by analgesia (0.5 mg/kg buprenorphine s. c.). The experiments were performed under isoflurane anesthesia (2.5% in 1.0 L of 100% O_2_ per min for induction and 2.5% in 1.0 L of 100% O_2_ per min for maintenance). Humane endpoints leading to euthanization were: maximum tumor diameter >1.5 cm, tumor exulceration, weight loss >15%, apathy, defense reaction when palpating tumors, respiratory problems, paresis, non-physiological body posture.

### Tumor model and experimental setup

Human BRAF V600E-positive melanoma cells (A375; ATCC CRL-1619, CLS Cell Lines Service GmbH, Eppelheim, Germany) were diluted in 0.1 mL of a 1:1 mixture of phosphate-buffered saline (PBS pH 7.4; GIBCO Life Technologies, Darmstadt, Germany) and Matrigel (BD Biosciences, San Jose, CA). The resulting mixture was subcutaneously injected into the left abdominal flank of n = 10 *Balb/c* nude mice (Charles River, Sulzfeld, Germany; 3 x 10^6^ cells/mouse). After reaching a tumor diameter of 0.5 cm, animals were randomized to either the therapy (n = 5) or the control group (n = 5). α_v_ß_3_-integrin-targeted optoacoustic imaging and magnetic resonance imaging (MRI) were performed on day 0 (baseline) and day 7 (follow-up). After optoacoustic imaging, animals were transferred to the MRI suite. T2-weighted (T2w) MRI was performed for anatomic colocalization and morphologic tumor response assessments (MR volumetry). Between baseline and follow-up imaging, animals were treated daily for one week with either BRAFi/MEKi combination therapy (BRAFi: encorafenib, 1.3 mg/kg/d; MEKi: binimetinib, 0.6 mg/kg/d; both Array BioPharma Inc., Boulder, CO) or a volume-equivalent placebo solution (1% carboxymethyl cellulose and 0.5% Tween-80 in ddH_2_0). After follow-up imaging, animals were sacrificed and the explanted tumors were fixed in formalin. Multiparametric immunohistochemical workup included ß_3_-integrin expression, microvascular density (CD31), and tumor cell proliferation (Ki-67).

### Optoacoustic imaging

Optoacoustic imaging was performed on a dedicated small animal imaging system (inVision 256-TF, iThera Medical GmbH, Munich, Germany) [17]. Briefly, a tunable optical parametric oscillator pumped by a neodymium-doped yttrium aluminum garnet laser provided excitation pulses with a duration of 9 ns at wavelengths from 680 nm to 980 nm at a repetition rate of 10 Hz with a wavelength tuning speed of 10 ms and a peak pulse energy of 100 mJ at 730 nm. Ten arms of a fiber bundle provided even illumination of a ring-shaped light strip of approximately 8 mm width. For ultrasound detection, 256 toroidally focused ultrasound transducers with a center frequency of 5 MHz (60% bandwidth), organized in a concave array of 270° angular coverage and a radius of curvature of 4 cm, were used. Animals were scanned at the following n = 11 wavelengths: 700 nm, 730 nm, 740 nm, 750 nm, 760 nm, 770 nm, 780 nm, 790 nm, 800 nm, 850 nm, 900 nm. N = 10 pulses were averaged per wavelength, giving a temporal resolution per multispectral cycle of 11 s. After animals were placed into the system, the positions of the rostral and caudal ends of the tumor were identified to mark the start and stop locations of the scan (typically approximately 1 cm). A step size of 0.5 mm was used to scan through the tumor. Animals were scanned before and 5 h after i. v. injection of a commercially available α_v_ß_3_-integrin-targeted fluorescent probe (IntegriSense 750, 4 nmol; Perkin Elmer, Waltham, MA). The data was reconstructed with ViewMSOT (iThera Medical GmbH, Munich, Germany) using the back projection method. Reconstructed baseline images were used to model the optoacoustic profile of the tumor using an adaptive match filter spectral unmixing algorithm [18]. These models were then applied on a per mouse basis to the 5 h scans, where deviations from the model consistent with the absorption spectrum of the α_v_ß_3_-integrin-targeted fluorescent probe were revealed. Regions of interest (ROI) were manually drawn on single wavelength images to identify the tumor, and the average spectrally unmixed α_v_ß_3_-integrin-targeted optoacoustic signal was quantified (arbitrary units, a. u.). Single wavelength background images were displayed in greyscale with a Frangi filter applied to enhance anatomical detail [19].

### MRI

MRI was performed on a clinical 3 Tesla scanner (MAGNETOM Skyra, Siemens Healthineers, Erlangen, Germany). Animals were scanned head-first in prone position. T2w data sets were acquired using a 2D Turbo Spin Echo sequence (TR = 5470 ms, TE = 91 ms, in-plane resolution 0.3 x 0.3 mm, matrix size 192 x 192, slice thickness 1.5 mm). MR volumetry was conducted on an external workstation using dedicated post-processing software written in-house (PMI; Platform for Research in Medical Imaging, version 0.4) [20].

### Planar whole-animal cryofluorescence imaging

In order to visualize the biodistribution of the targeted fluorescent probe, planar cryofluorescence images were acquired as described elsewhere [21]. Briefly, a cryostat was retrofitted with a light source, filters, and a camera which enabled acquisition of color images as well as fluorescence images of frozen specimen.

### In vivo blocking studies

Competitive in vivo blocking studies were performed to confirm the target specificity of the α_v_ß_3_-integrin-targeted fluorescent probe. For blocking, α_v_ß_3_-integrin receptor antagonist cilengitide (800 μg; Selleck Chemicals, Houston, TX) was injected i. v. 15 min prior to injection of the α_v_ß_3_-integrin-targeted fluorescent probe (4 nmol). Optoacoustic imaging and signal quantification were then performed as described above and signal intensities of blocked and unblocked animals were compared accordingly.

### Immunohistochemistry

Microscope slides with 3 μm sections from paraffin-embedded tissue were dewaxed and rehydrated following standard procedures (preheating at 60°C, xylene substitute [Neo-Clear, Merck KgaA, Darmstadt, Germany] graded series of ethanol (100%, 96%, 80% and 70%), followed by double distilled water). After antigen demasking (microwave irradiation at 600 W, 0.1 M citrate buffer pH 6.0) and overnight incubation with the primary antibodies (anti-ß_3_-integrin antibody Abcam ab179473, 1:500, anti-CD31 antibody Abcam ab28364 1:50, anti-Ki-67 antibody SP6 Abcam ab16667 1:100 [all Cambridge, United Kingdom]) at 4°C, tissue samples were further processed using the EnVision+ System HRP (DAB or AEC) (DAKO Diagnostika, Hamburg, Germany) kit according to the manufacturer´s instructions. Slides were counterstained with Mayer´s Haemalaun (Merck KgaA, Darmstadt, Germany) and covered with Kaiser´s Glycerin Gelatine (Merck KgaA, Darmstadt, Germany). The optical density of ß_3_-integrin expression was measured in ten random fields at 200x magnification using ImageJ (“Fiji” version, www.fiji.sc). CD31-positive microvessels and Ki-67-positive nuclei were quantified in ten random high-power fields at 200x magnification.

### Statistical analysis

Statistical analysis was performed using commercially available statistics software (SPSS 24, IBM Corp., Armonk, NY). The continuous parameters were expressed as means with standard deviations (95% confidence intervals). For intragroup comparisons (follow-up vs. baseline), the Wilcoxon test was applied, while the Mann-Whitney U test was applied for intergroup comparisons. The confirmatory tests (α_v_ß_3_-integrin-specific optoacoustic signal at follow-up vs. baseline; ß_3_-integrin in the therapy vs. the control group) were compared against Bonferroni-Holm-adjusted α’ levels (α = 0.05). All other tests were compared against α = 0.05 as they were considered exploratory.

## Results

### Optoacoustic imaging

**Fig 1:**
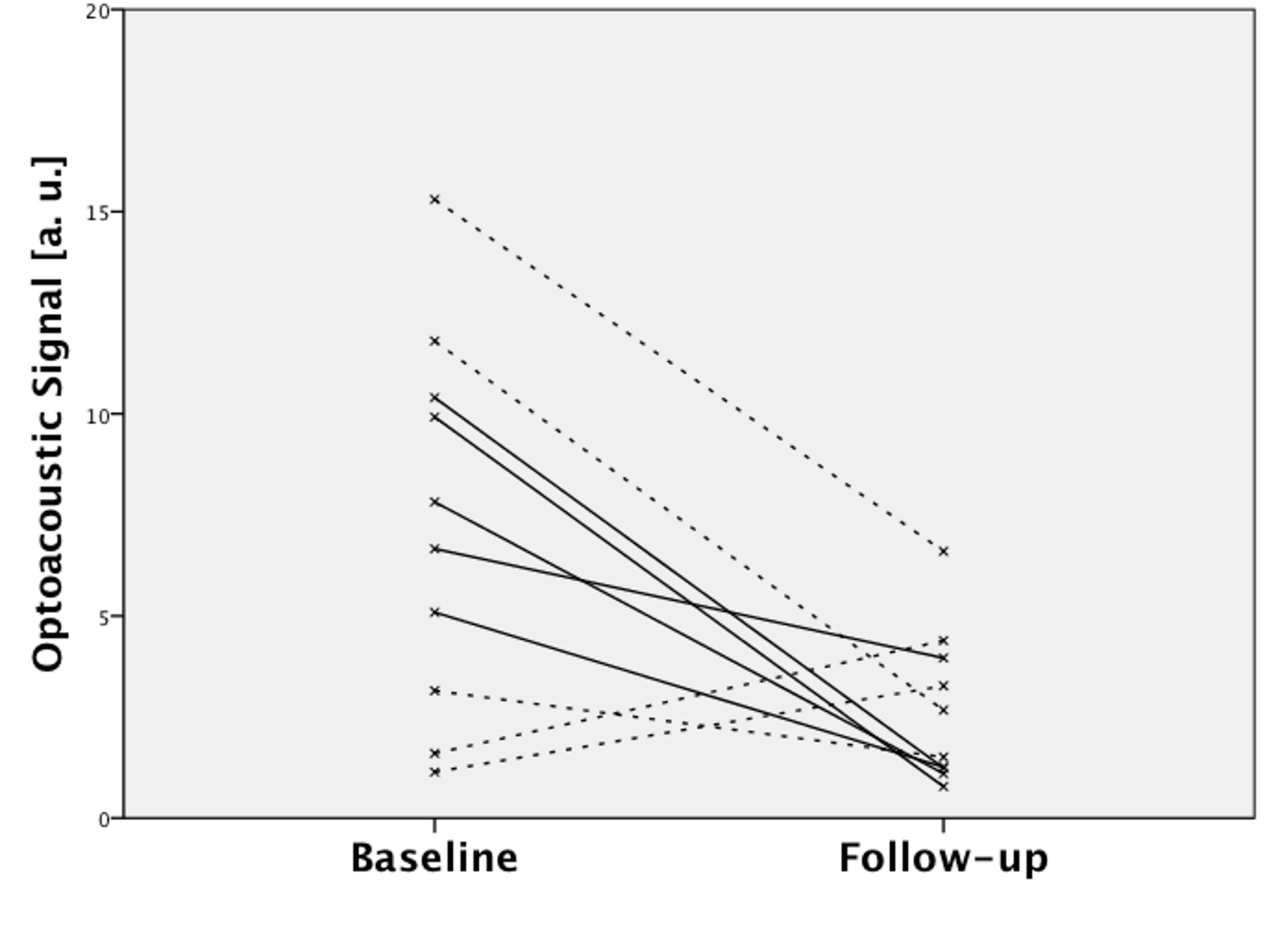
Optoacoustic signal intensities at baseline and follow-up. Solid lines: therapy group. Dashed lines: control group. Note the unidirectional signal decrease in the therapy group and the omnidirectional development of signal intensities in the control group.

**Fig 2:**
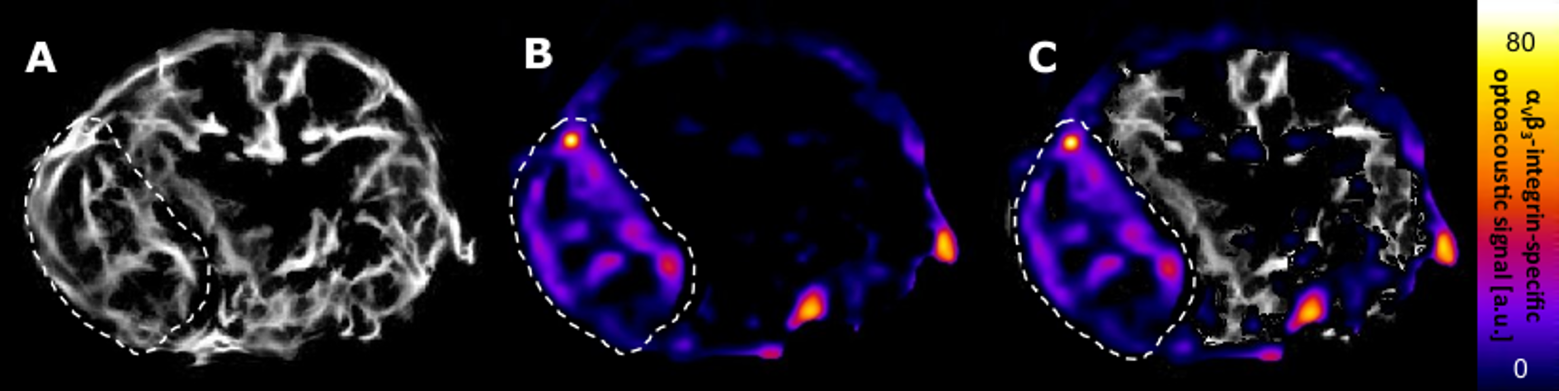
Overview of α_v_ß_3_-integrin-specific optoacoustic imaging. *Balb/c* nude mouse. Axial plane. Tumor margins indicated by white dashed line. A: Optoacoustic image (850 nm). B: Spectrally unmixed α_v_ß_3_-integrin-specific signal. C: Overlay of A and B. Note the tumor-specific binding of the α_v_ß_3_-integrin-targeted fluorescent probe.

**Fig 3:**
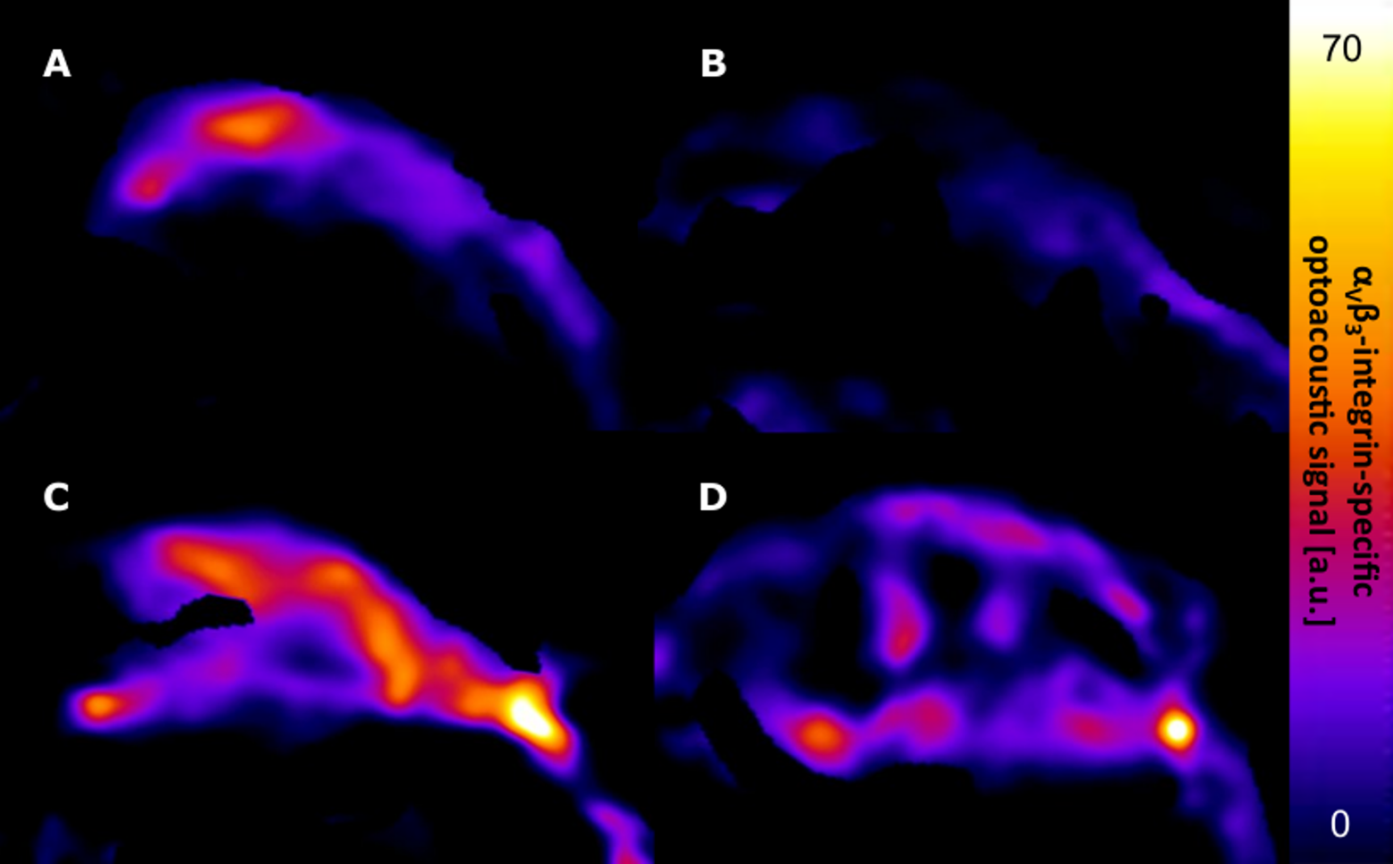
α_v_ß_3_-integrin-specific signal before and after treatment of *Balb/c* nude mice. A: therapy, baseline. B: therapy, follow-up. C: control, baseline, D: control, follow-up. Note the significant decrease in α_v_ß_3_-integrin-specific optoacoustic signal following BRAFi/MEKi combination therapy (B vs. A). No significant change in α_v_ß_3_-integrin-specific optoacoustic signal is observed in the control group (D vs. C).

**Fig 4:**
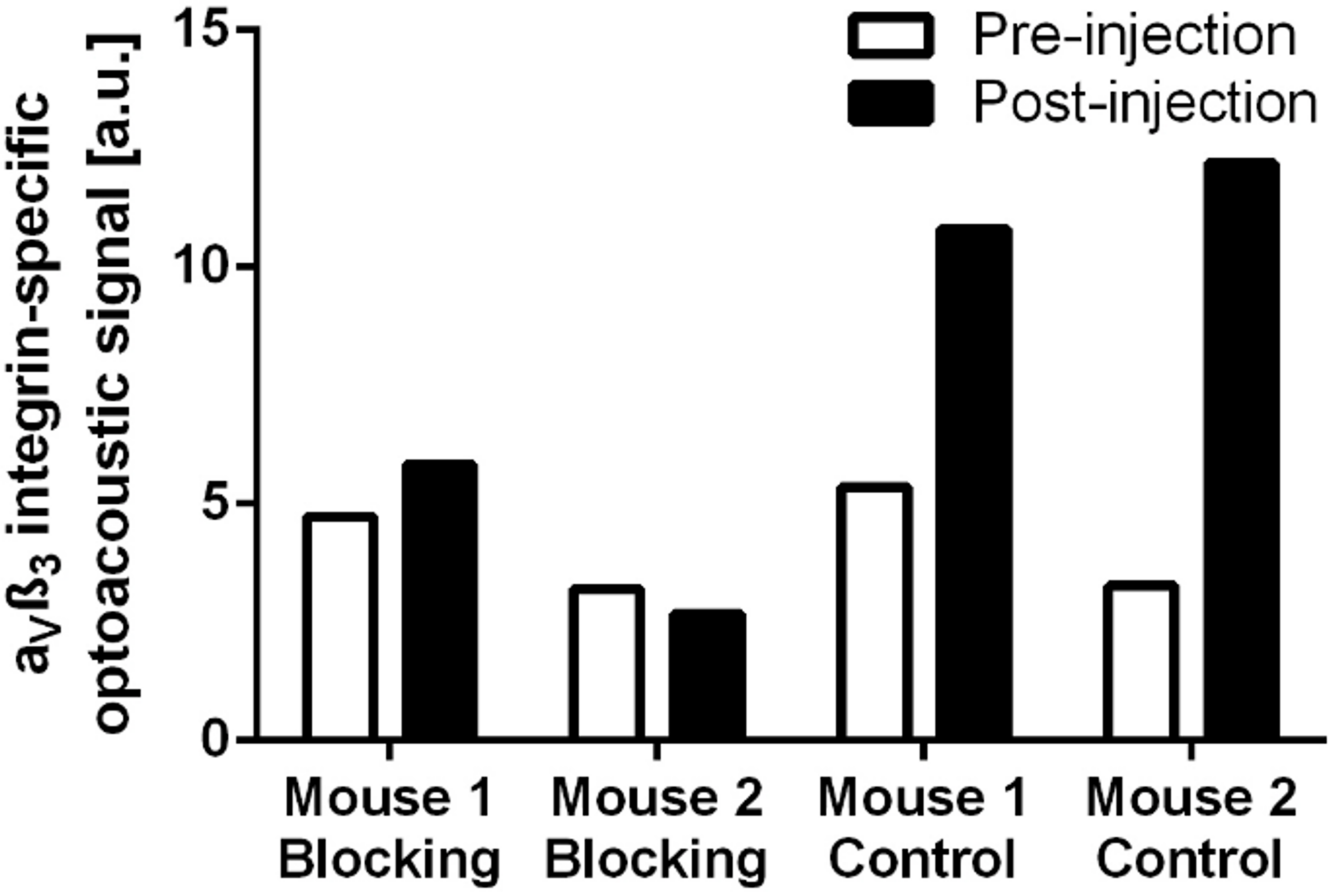
In vivo blocking experiments. Competitive in vivo blocking studies with α_v_ß_3_-integrin receptor antagonist cilengitide confirmed the specificity of the targeted fluorescent probe. Note the significant optoacoustic tumor signal increase in unblocked animals (control). No significant change in optoacoustic tumor signal was observed in blocked animals.

**Fig 5:**
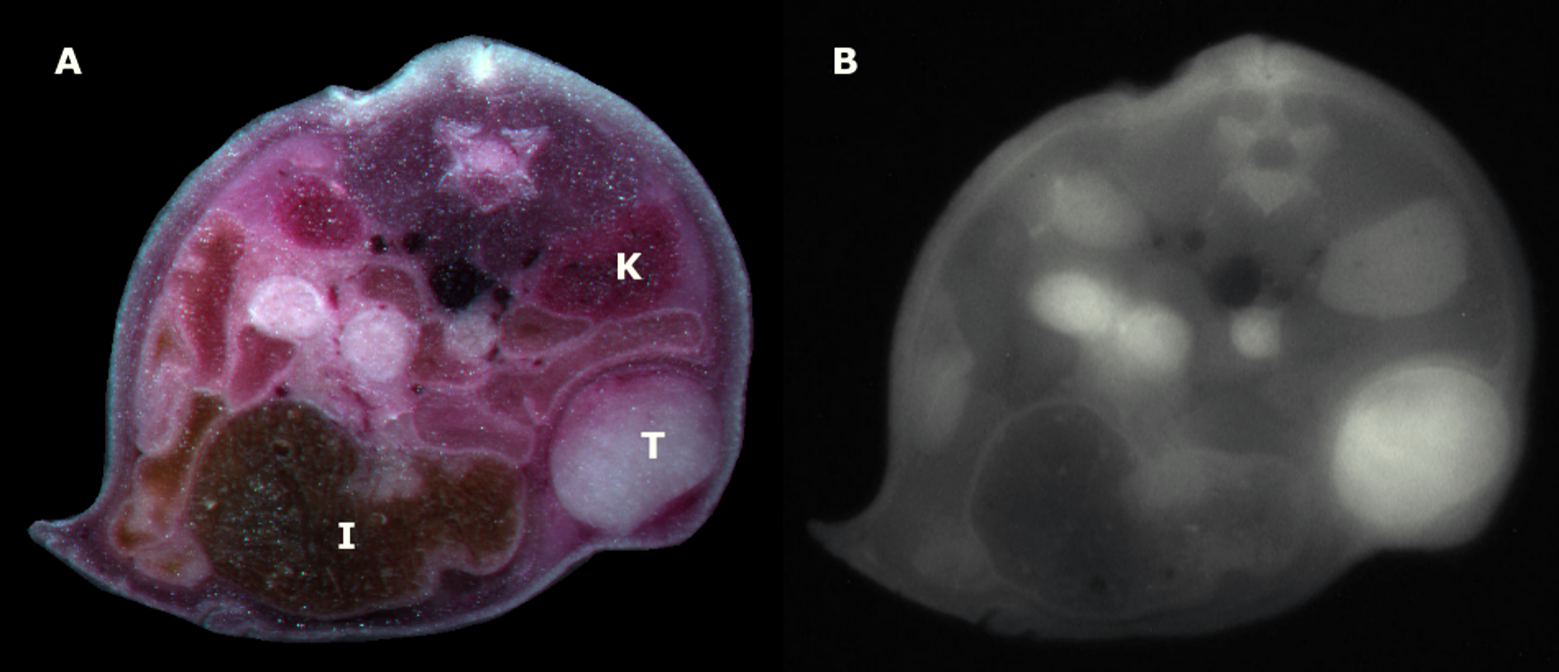
Planar whole-animal cryofluorescence imaging. *Balb/c* nude mouse. A: macroscopic color image. B: fluorescence image. T: tumor; K: kidneys; I: intestines. The targeted fluorescent probe showed a tumor-specific binding. Analogous to the in vivo optoacoustic images, little background signal is noted in the intestines.

The α_v_ß_3_-integrin-specific optoacoustic signal was significantly reduced under therapy, demonstrating a unidirectional decline in all animals (mean signal: from 7.98±2.22 a. u. to 1.67±1.30 a. u.; p = 0.043). No statistically significant change of the optoacoustic signal was observed in the control group, with a heterogeneous development of individual values (mean signal: from 6.60±6.51 a. u. to 3.67±1.93 a. u.; p = 0.500). There was no statistically significant difference in baseline optoacoustic signals between the therapy and the control group (mean signal: therapy 7.98±2.22 a. u. vs. control 6.60±6.51 a. u.; p = 0.690). Individual optoacoustic signal intensities at baseline and follow-up are displayed in Table 1 and Fig 1. Fig 2 shows a single wavelength optoacoustic image for anatomical reference in greyscale and the spectrally unmixed, α_v_ß_3_-integrin-specific signal in fire. Representative color-coded tumor maps of exemplary animals from the therapy and the control group before and after treatment are provided in Fig 3. Competitive blocking studies confirmed the target specificity of the α_v_ß_3_-integrin-targeted fluorescent probe (Fig 4). The α_v_ß_3_-integrin-targeted fluorescent probe demonstrated tumor-specific binding in all animals with a representative biodistribution of the fluorescent probe shown in Fig 5.

**Table 1:** Optoacoustic tumor signals and tumor volumes at baseline and follow-up. Optoacoustic signal intensities displayed in [a. u.]. Volumes (Vol) displayed in [mm^3^].

Animal no.	T/C*	Signal_Baseline_	Signal_Follow-Up_	Vol_Baseline_	Vol_Follow-Up_	ΔVol
1	T	10.40	1,24	23.5	190.7	167.7
2	T	9.92	0.78	32.0	143.4	111.4
3	T	7.82	1.10	51.1	144.8	93.7
4	T	5.09	1.26	38.6	88.1	49.5
5	T	6.66	3.96	30.0	144.2	114.2
Mean±SD^†^	T	7.98±2.22	1.67±1.30	35±10	142±36	107±43
6	C	15.30	6.60	117.1	244.6	127.5
7	C	11.80	2.67	60.4	120.4	60.0
8	C	3.15	1.51	57.4	205.7	148.3
9	C	1.14	3.27	43.5	197.3	153,8
10	C	1.60	4.39	29.4	101.8	72,4
Mean±SD^†^	C	6.60±6.51	3.67±1.93	62±33	174±54	112±39

*: T = therapy group; C = control group

†: SD = standard deviation

### MRI

**Fig 6:**
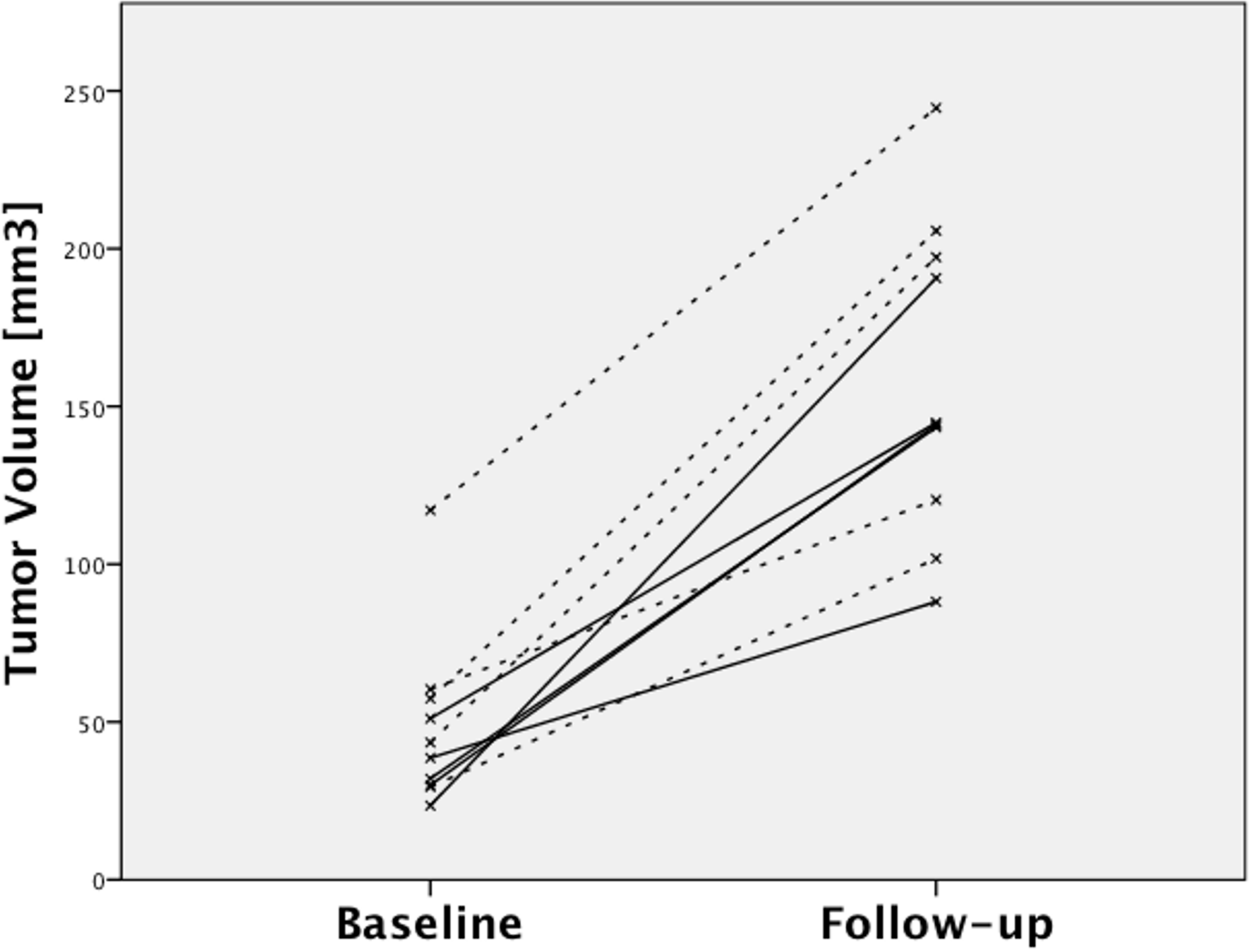
Tumor volumes at baseline and follow-up. Solid lines: therapy group. Dashed lines: control group. In both therapy and control group, tumor volumes increased over the treatment course with no significant intergroup difference.

**Fig 7:**
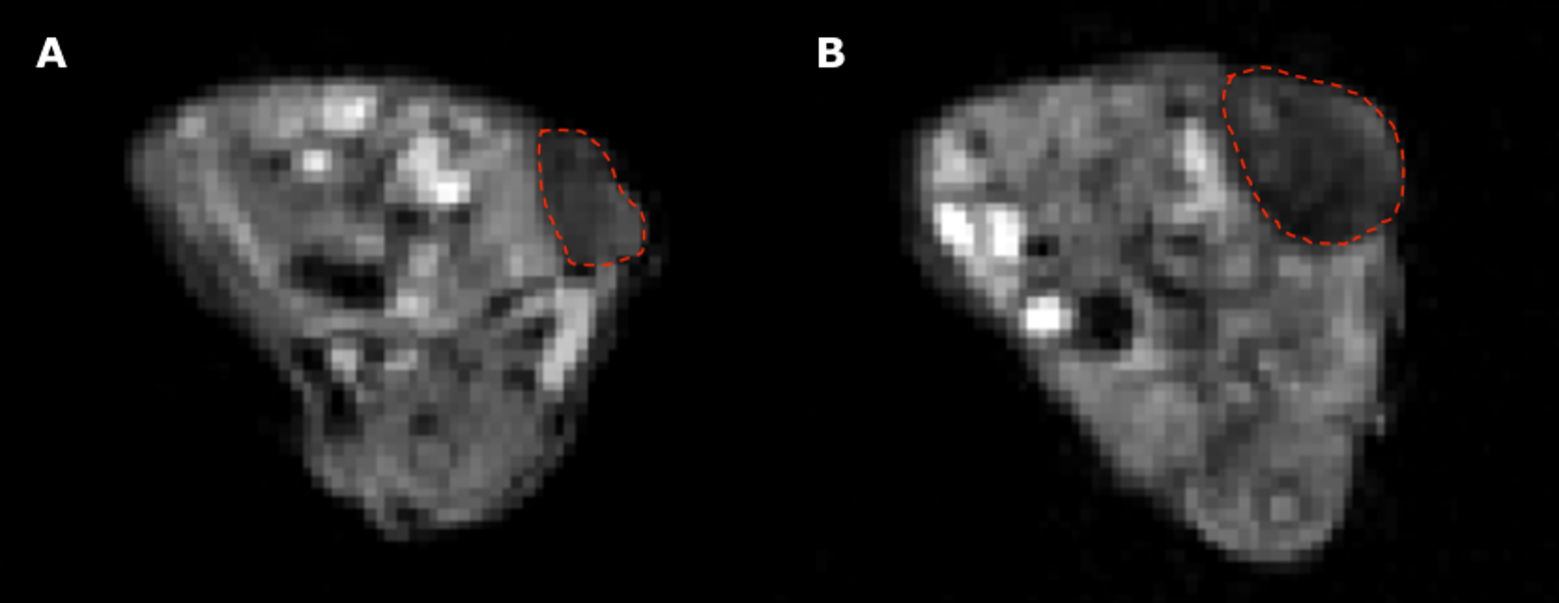
Morphologic MRI data sets of a representative animal from the control group at baseline and follow-up. ***Balb/c* nude mouse.** A: baseline. B: follow-up. Tumor indicated by red dashed line. Note the tumor volume increase from baseline to follow-up.

Tumor volumes increased in both the therapy and the control group with no statistically significant intergroup difference (ΔVol_Therapy_ +107±42 mm^3^ vs. ΔVol_Control_ +112±44 mm^3^; p = 0.841). There was no difference in baseline tumor volumes between the therapy and the control group (Vol_TherapyBL_ 35±10 mm^3^ vs. Vol_ControlBL_ 62±33 mm^3^; p = 0.151). Individual tumor volumes at baseline and follow-up are provided in Table 1 and Fig 6. T2w images of a representative animal from the control group at baseline and follow-up are provided in Fig 7.

### Immunohistochemistry

**Fig 8:**
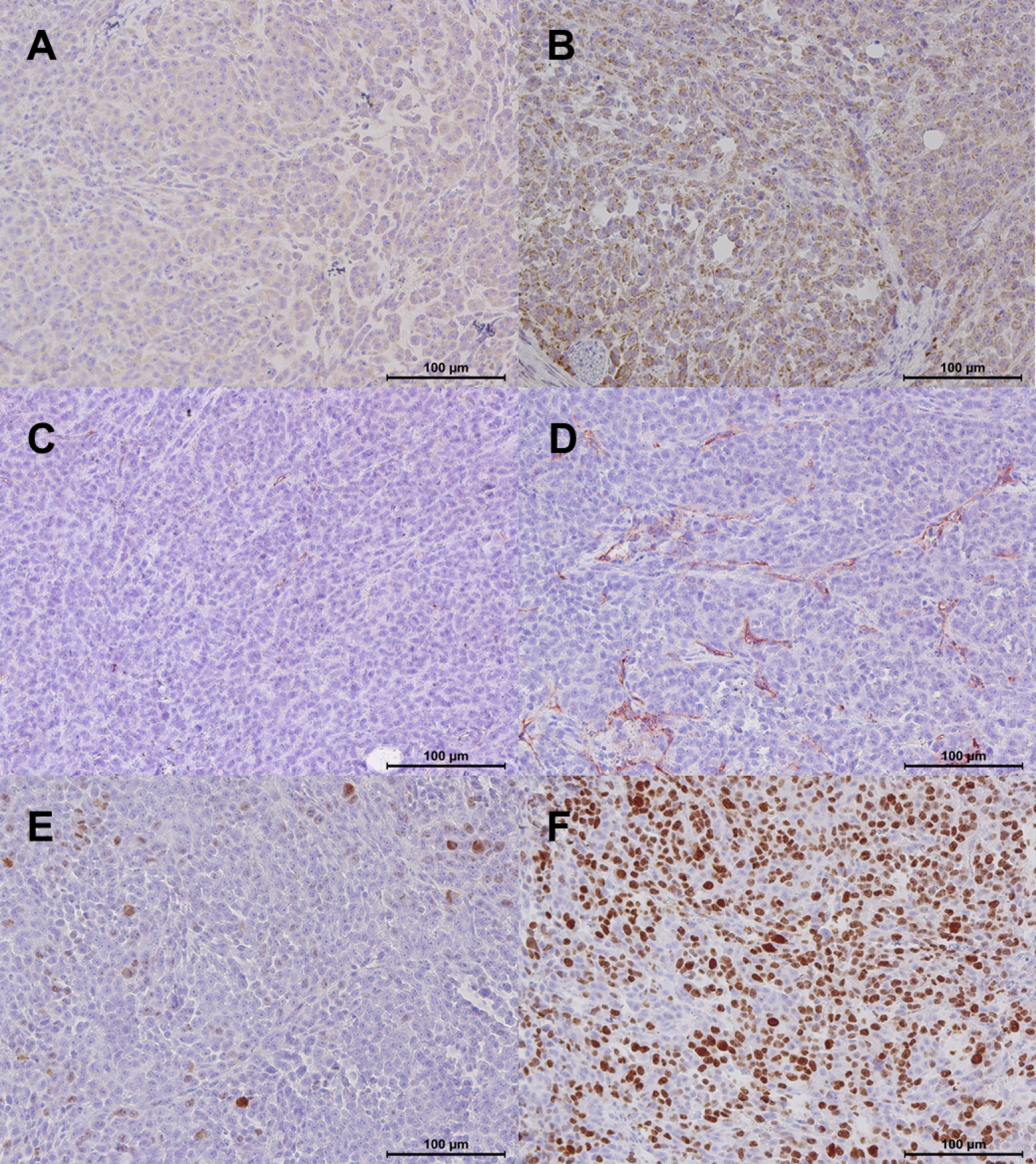
Immunohistochemical stainings of representative tumor sections from the therapy and the control group. A: ß_3_-integrin, therapy. B: ß_3_-integrin, control. C: CD31, therapy. D: CD31, control. E: Ki-67, therapy. F: Ki-67, control. Note the lower ß_3_-integrin expression (A vs. B; p = 0.008), microvascular density (CD31, C vs. D; p = 0.008), and tumor cell proliferation (Ki-67, E vs. F; p = 0.151) in the therapy group compared to the control group. Magnification: 200x.

In line with the optoacoustic imaging results, ß_3_-integrin expression was statistically significantly lower in the therapy group (ß_3_: 0.20±0.02 vs. 0.39±0.05; p = 0.008). We observed a statistically significantly lower microvascular density in the therapy group compared to the control group (CD31: 119±15 vs. 292±49; p = 0.008). There was a statistically non-significant lower tumor cell proliferation in treated compared to untreated animals (Ki-67: 3,925±1,693 vs. 5,782±1,092 p = 0.151). Individual values for the immunohistochemical parameters are provided in Table 2. Immunohistochemical stainings of representative tumor sections are provided in Fig 8.

**Table 2:** Individual immunohistochemical parameters.

Animal no.	T/C*	ß_3_-Integrin	CD31	Ki-67
1	T	0.172	133	2,937
2	T	0.178	123	2,587
3	T	0.209	123	2,713
4	T	0.205	93	6,389
5	T	0.218	121	5,001
Mean±SD^†^	T	0.20±0.02	119±15	3,925±1,693
6	C	0.358	300	4,623
7	C	0.475	254	6,508
8	C	0.371	371	4,700
9	C	0.352	282	6,000
10	C	0.402	252	7,078
Mean±SD^†^	C	0.39±0.05	292±49	5,782±1,092

*: T = therapy group; C = control group

†: SD = standard deviation

## Discussion

In the present study, we investigated α_v_ß_3_-integrin-targeted optoacoustic imaging and morphologic MRI for the non-invasive in vivo monitoring of a BRAFi/MEKi combination therapy in a murine model of human melanoma. Imaging was validated in and ex vivo: (1) In vivo blocking studies verified the α_v_ß_3_-integrin specificity of the targeted fluorescent probe, (2) ex vivo planar whole-animal cryofluorescence imaging confirmed tumor-specific probe binding, and (3) ex vivo immunohistochemistry demonstrated a statistically significant suppression of ß_3_-integrin expression under therapy. In correlation with the ex vivo immunohistochemistry, the α_v_ß_3_-integrin-targeted optoacoustic signal was statistically significantly reduced under therapy, while no statistically significant change was observed in the control group. Tumor volumes increased in both the therapy and the control group. This proof-of-principle study demonstrates the feasibility of optoacoustic imaging with a targeted fluorescent probe for the longitudinal, quantitative dual time point monitoring of a molecular cancer therapy in vivo, adding complementary molecular information on α_v_ß_3_-integrin receptor status to morphology-based assessments of tumor response.

Several groups investigated the potential of preclinical optoacoustic imaging with targeted probes for the non-invasive characterization of the tumor microenvironment in vivo [16, 22–25]. α_v_ß_3_-integrin-targeted fluorescent probes were studied to qualitatively visualize experimental human breast carcinomas and glioblastomas [16, 24]. However, reproducible signal quantifiability is an essential prerequisite for therapy monitoring. Our study adds to the literature as it presents a first approach to quantify the fluorescent signal at different time points under therapy validated by immunohistochemistry, evaluating the potential of optoacoustic imaging for therapy monitoring in future clinical trials. Optoacoustic imaging yields high translational potential with a focus on tumors located close to the body surface. Clinical optoacoustic imaging systems were already investigated in melanoma, inflammatory bowel disease, breast cancer, and thyroid cancer [26–29]. Clinical optoacoustic imaging allows for the acquisition of anatomical and functional information on tumors and healthy tissues exploiting intrinsic tissue contrast and visualizing endogenous absorbers (melanin and oxygenated/deoxygenated hemoglobin) [26, 27]. However, in order to generate molecular information such as tumor receptor status, exogenous absorbers, i. e., targeted probes, are necessary [30]. Despite the promising preclinical results reported in the literature, data on clinical optoacoustic imaging with targeted fluorescent probes are still lacking and data on the use of targeted fluorescent probes in humans are limited. Indocyanine green and methylene blue are two non-targeted near-infrared range fluorochromes approved by the Food and Drug Administration for the use in humans, but these unspecific blood pool agents fail to provide information on tumor-inherent target structures [31]. In clinical studies, targeted fluorescent agents were already investigated for the guidance of surgical and endoscopic procedures [32–34]. For instance, a folate-receptor α-targeted fluorescent probe emitting in the visible spectrum (500 nm) was recently investigated in patients with ovarian or breast cancer to intraoperatively detect additional tumor lesions and to aid the definition of tumor margins [33]. Contrast agents that absorb in near-infrared, which would enable the deepest possible imaging in tissue for optoacoustics, are also available and were applied in clinical studies for optical imaging. For example, bevacizumab-IRDye800CW was used to detect colorectal peritoneal metastases intraoperatively with the use of a fluorescence camera [35]. Optoacoustic imaging could be used for similar applications, though its capability of deeper tissue penetration could potentially offer additional benefits intraoperatively or in non-invasive settings. However, biodistribution and biocompatibility of targeted, near-infrared fluorescence imaging agents in humans remain major issues that need to be addressed before clinical translation. Binding to non-target tissues as well as the enhanced permeability and retention effect may lower the target-to-background signal and therefore limit the applicability for therapy monitoring [31, 36]. For local staging under therapy, subcutaneous injection of the targeted probe analogously to interstitial MR lymphography with consecutive lymphatic transport to the target tumor (melanoma) would be a possible approach to reduce the systemic side effects in humans and may be investigated in future studies.

The role of optoacoustic imaging compared to alternative α_v_ß_3_-integrin-targeted imaging modalities remains to be defined. Positron emission tomography (PET) with targeted radionuclides as well as MRI using targeted contrast agents also allow for the non-invasive characterization of α_v_ß_3_-integrin receptor status in vivo [13, 37–39]. Compared to optoacoustic imaging, these modalities are not limited by low tissue penetration depth and are superior with regard to whole-body tumor staging. However, the lack of ionizing radiation, lower costs compared to PET and MRI, and the availability of clinical imaging devices for bedside use are major advantages of optoacoustic imaging. One potential application of handheld optoacoustic scanners may be the monitoring of superficially-located target lesions, i. e., lymph node metastases, cutaneous metastases, or the primary tumor (if unresectable). This could be performed as part of a multimodality imaging protocol complementary to established imaging modalities such as ^18^F-fluorodeoxyglucose-PET/computed tomography or MRI, generating a surrogate of whole-body tumor receptor status under targeted therapy.

### Limitations

We acknowledge several limitations of the present study. First, the animal number was small and the results including reproducibility of the quantitative data need to be validated in larger cohorts. However, this can be considered appropriate for the study purpose, which was to provide a first proof-of-principle of quantitative dual time point optoacoustic imaging for therapy monitoring in a small animal model of human melanoma. In addition, Joseph et al. recently provided evidence for the reproducibility and repeatability of quantitative in vivo optoacoustic imaging [40]. Second, the imaging results were validated by single time point immunohistochemistry and the follow-up interval was limited to one week. Additional follow-up intervals for both imaging and immunohistochemistry may reveal deeper insights into the development of α_v_ß_3_-integrin expression and the according optoacoustic signal over the therapy course. Third, α_v_ß_3_-integrin-targeted imaging may be limited by elevated background signal as α_v_ß_3_-integrin is not only expressed by tumors, but also several non-target tissues including the gut and the liver [41]. The localization of the tumor may thus have an effect on ROI selection and signal quantifiability. Nevertheless, the detected α_v_ß_3_-integrin-specific optoacoustic signal allowed for a good depiction of the tumor against the background in all animals, which can be attributed to the relatively high α_v_ß_3_-integrin expression of the A375 cell line compared to other available melanoma models [42].

### Conclusions

In this experimental study, α_v_ß_3_-integrin-targeted optoacoustic imaging allowed for the in vivo monitoring of a BRAFi/MEKi combination therapy in a murine model of human melanoma. Our results provide a proof-of-principle of quantitative dual time point optoacoustic imaging for therapy monitoring in vivo.
